# Mastoid cavity obliteration leads to a clinically significant improvement in health-related quality of life

**DOI:** 10.1007/s00405-020-05881-4

**Published:** 2020-03-06

**Authors:** Nora M. Weiss, David Bächinger, Jannik Botzen, Wilma Großmann, Robert Mlynski

**Affiliations:** 1 Department of Otorhinolaryngology, Head and Neck Surgery “Otto Körner”, Rostock University Medical Center, Doberaner Strasse 137-139, 18057 Rostock, Germany; 2 Department of Otorhinolaryngology, Head and Neck Surgery, University Hospital Zurich, Zurich, Switzerland; 3 University of Zurich, Zurich, Switzerland

**Keywords:** Chronic middle ear disease, Chronic otitis media, Cholesteatoma, Mastoid cavity, Obliteration, ZCMEI-21

## Abstract

**Objective:**

To assess the change in health-related quality of life (HRQoL) in patients undergoing mastoid cavity obliteration.

**Methods:**

Patients who had undergone canal wall-down mastoidectomy for chronic otitis media with creation of a persistent mastoid cavity and underwent revision tympanomastoid surgery including mastoid cavity obliteration using autologous material were included. Audiological measurements including air conduction (AC) and bone conduction (BC) pure-tone averages (PTA) and the air–bone gap (ABG) were assessed. Health-related Quality of Life (HRQoL) was assessed by the Zurich Chronic Middle Ear Inventory (ZCMEI-21) pre- and postoperatively.

**Results:**

A total of 25 patients (16 females and 9 males; mean age 51.6 years, 14 right and 11 left ears) were included. Patients were reexamined after a mean follow-up period of 9.2 months (SD = 6.5) after obliteration of the mastoid cavity. Compared to the preoperative visit, patients showed a significantly reduced AC PTA at the postoperative visit (mean difference: − 4.1; SD = 10.4, *p* = 0.045). The mean ZCMEI-21 score changed from 31.7 (SD = 14.5) preoperatively to 17.4 (SD = 15.1) postoperatively (mean difference: − 14.3; SD = 19.1; *p* = 0.0002). The mean ZCMEI-21 score changes were neither correlated to the AC PTA shift (*p* = 0.60) nor to the ABG shift (*p* = 0.66).

**Conclusions:**

This is the first study reporting a highly significant and clinically important improvement in HRQoL after mastoid cavity obliteration in a prospective setting. The improvement in HRQoL was not correlated to the hearing improvement. As a clinical implication, we provide evidence for a substantial subjective benefit of the surgical obliteration of a symptomatic mastoid cavity and, therefore, encourage this surgical procedure.

## Introduction

Historically, in extended inflammation processes of the middle ear and mastoid, an open mastoid cavity was created without reconstruction under the aim of draining the disease into the bony outer ear canal [1]. Nowadays, the primary creation of a mastoid cavity is performed as part of a canal wall-down mastoidectomy in cases of large cholesteatoma or inflammatory processes inside the mastoid [2]. Depending on the extent of the mastoid cavity and the size of the external auditory canal, the self-cleaning process of the mastoid cavity may be disturbed, leading to recurrent infections, secretion, vertigo, hearing impairment, and frequent consultation of an ENT specialist [3]. In these cases, the treatment of choice consists of the secondary surgical obliteration of the mastoid cavity as first described by Mosher in 1911 [4]. Multiple methods and materials for cavity obliteration, which is commonly performed in combination with meatoplasty, have been developed and tested [5, 6]. Usually, the use of autologous material is preferred due to its good biocompatibility. Cartilage, either from the concha, tragus or nasal septum, muscle flaps, bone pâté or fascia are used. Drawback of autologous material may be natural shrinkage and the limited availability of after repeated revision surgery [7]. Additionally, several xenografts and alloplastic materials have been designed and tested in clinical and experimental settings [8–10]. However, the mechanism of biomaterial-based regeneration processes has still to be understood and recent work concerning allogeneic materials showed an insufficient cavity obliteration and high rates of revision surgery [11].

In cases of a persistent mastoid cavity, symptoms such as chronic ear discharge, ear pain, and hearing impairment may severely impair patient’s health-related quality of life (HRQoL). Furthermore, it has been reported that hearing impairment is a major risk factor for the development of dementia and cognitive dysfunction [12]. It has to be assumed that the patient’s subjective benefit from tympanomastoid surgery is not only determined by the postoperative reduction of the air–bone gap (ABG) [13]. Therefore, focusing on the audiological outcome alone may only insufficiently assess the surgical results [14]. Moreover, patient-reported outcome measures are being used increasingly and have gained importance to measure therapeutic success [15, 16]. Usually, standardized questionnaires investigating everyday life situations, difficulties in communication or social contacts, and co-symptoms like tinnitus are used to assess the impairment due to hearing handicap [17, 18]. The Glasgow benefit inventory (GBI) is used to report the subjective success of otosurgical interventions [19, 20]. However, disadvantages of the GBI include the exclusively retrospective application, and therefore, changes in HRQoL cannot be reported reliably. In contrast, the Zurich Chronic Middle Ear Inventory-21 (ZCMEI-21) was designed as a disease-specific instrument assessing disease-specific symptoms and their impact on quality of life in chronic otitis media (COM) [11, 15, 21–23]. The responsiveness to change of the ZCMEI-21 has been recently investigated, thus increasing its clinical utility and facilitating comparisons among different surgical interventions [24].

To our knowledge, HRQoL in patients undergoing mastoid cavity obliteration has never been studied in a prospective setting using a disease-specific HRQoL instrument. In the past, conflicting results about the change in quality of life have been obtained using generic questionnaires (e.g., the GBI) in three small retrospective cohort studies [25–27]. Whereas two studies reported a subjective benefit from surgery [25, 26], another study reported a majority of patients experiencing no change in quality of life after mastoid obliteration [27]. Therefore, the aim of this study was to systematically investigate the change in HRQoL in patients undergoing mastoid cavity obliteration in a prospective setting using a disease-specific instrument.

## Methods

### Ethical consideration

The study protocol was approved by the local Ethics Committee in accordance with the Helsinki declaration (Registration-number: A2017-0101). Informed consent was obtained from all the participants.

### Study design and patient selection

In this prospective follow-up study, consecutive adult patients receiving mastoid cavity obliteration were assessed for inclusion between July 2017 and October 2019 in a tertiary referral center of a university hospital. The main inclusion criterium was a preexisting mastoid cavity after mastoidectomy using a canal wall-down technique without primary reconstruction. Patients undergoing cholesteatoma surgery and cavity obliteration or canal reconstruction within the same surgery were excluded. Autologous reconstruction material (local pedicled muscle flaps, bone paté, temporal muscle fascia, and cartilage) was used to obliterate the open mastoid cavity and/or to reconstruct the posterior canal wall. In cases with persistent ABG, ossiculoplasty was performed to improve the hearing. Patients completed preoperative and postoperative study visits. Patients included in the study underwent pure-tone audiometry and completed the ZCMEI-21 questionnaire at both visits. No children were included in the study.

### Audiometric assessment

All audiometric measurements were performed with calibrated instruments in a sound-proof room (DIN EN ISO 8253) by audiologically trained staff. Measurements included standard pure-tone audiometry, performed with a clinical audiometer (AT1000, Auritec, Hamburg, Germany) in 5 dB steps. The ABG was calculated as the difference between the pure-tone average (PTA) of the air conduction (AC) PTA measured at 0.5, 1, 2, and 3 kHz (PTA_0.5–3 kHz_) and the respective bone conduction (BC) PTA. According to recommendations in hearing reporting standard [28] and to the Committee on Hearing Equilibrium guidelines [29], the ABG_0.5–3 kHz_ was chosen for evaluating the results of treating conductive hearing loss. Therefore, only the ABG_0.5–3 kHz_ was further analyzed and is referred to as ABG. Audiometry was performed pre- and postoperatively.

### ZCMEI-21 Questionnaire

The ZCMEI-21 was used to assess HRQoL [15]. The ZCMEI-21 as a disease-specific questionnaire for chronic middle ear disease has been translated in several languages [21, 22, 30] and is used in clinical settings for research and clinical practice [11, 15]. The ZCMEI-21 consists of four subscales concerning ear signs and symptoms, hearing function, psychosocial impact, and the use of medical resources. Answers are presented using a five-point Likert-scale. Higher scores indicate a poorer quality of life [15] and the minimal clinically important difference (MCID) is estimated to 5 [24]. The ZCMEI-21 was designed as a disease-specific instrument to assess HRQoL in patients suffering from chronic middle ear disease and may also be used after surgical interventions. The ZCMEI-21 was completed prior surgery and at the follow-up visit after surgery.

### Statistical analysis

All statistical tests were selected before data collection. Statistical analyses were performed using Microsoft Excel (version 15.29, Microsoft Corporation, Redmond, WA, USA) and Prism (version 8, GraphPad Software, La Jolla, CA, USA). The significance level was set to *p* < 0.05. The assumption of normality in AC and ABG distributions was tested graphically using quantile–quantile plots and with the Kolmogorov–Smirnov test. If not otherwise specified, data are presented as mean with standard deviation (SD) or absolute numbers with percentages. A paired sample Student’s *t* test was performed to compare pre- and postoperative means in audiometric outcomes and ZCMEI-21 scores. Correlations were calculated using Pearson’s correlation coefficient.

## Results

A total of 31 patients receiving secondary tympanomastoid surgery using autologous reconstruction material between July 2017 and October 2019 were assessed for inclusion. Postoperative data were available of 25 patients (16 females and 9 males; mean age 51.6 years, 14 right and 11 left ears) and were analyzed. The mean time period between the obliteration of the open mastoid cavity and the postoperative follow-up visit was 9.2 months (SD = 6.5). At the postoperative visit, the mastoid cavity was sufficiently obliterated and observable in 24 cases (96%). One patient (4%) underwent revision surgery due to a necrotic muscle flap 1 month after obliteration surgery.

### Audiological outcomes

**Fig. 1:**
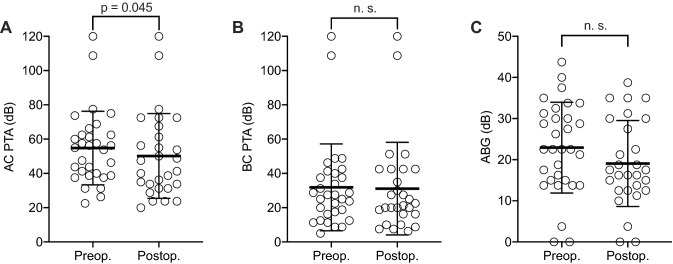
Audiometric outcomes after mastoid cavity obliteration. Pre- and postoperative air conduction (AC) (**a**) and bone conduction (BC) (**b**) pure-tone average (PTA) as well as the air–bone gap (ABG). The bold line represents the mean; error bars indicate standard deviation

Compared to the preoperative visit, patients showed a statistically significant reduction in AC threshold at the postoperative follow-up visit (mean difference: − 4.1 dB; SD = 10.4, *p* = 0.045; Fig. 1a). No significant difference was found between the pre- and postoperative BC thresholds (mean difference: − 1.8 dB; SD = 7.1, *p* = 0.19; Fig. 1b) and the ABG (mean difference: − 2.32; SD = 9.32; *p* = 0.20; Fig. 1c). Only one patient (4%) showed a clinically relevant deterioration of hearing (defined as > 10 dB AC) and six patients (24%) had an improvement of hearing of more than 10 dB AC.

### Medical resources

The number of consultations in the past 6 months of an ENT doctor decreased from a mean of 4.0 (SD = 2.3) consultations to 2.0 (SD = 2.6).

### Health-related quality of life

**Fig. 2:**
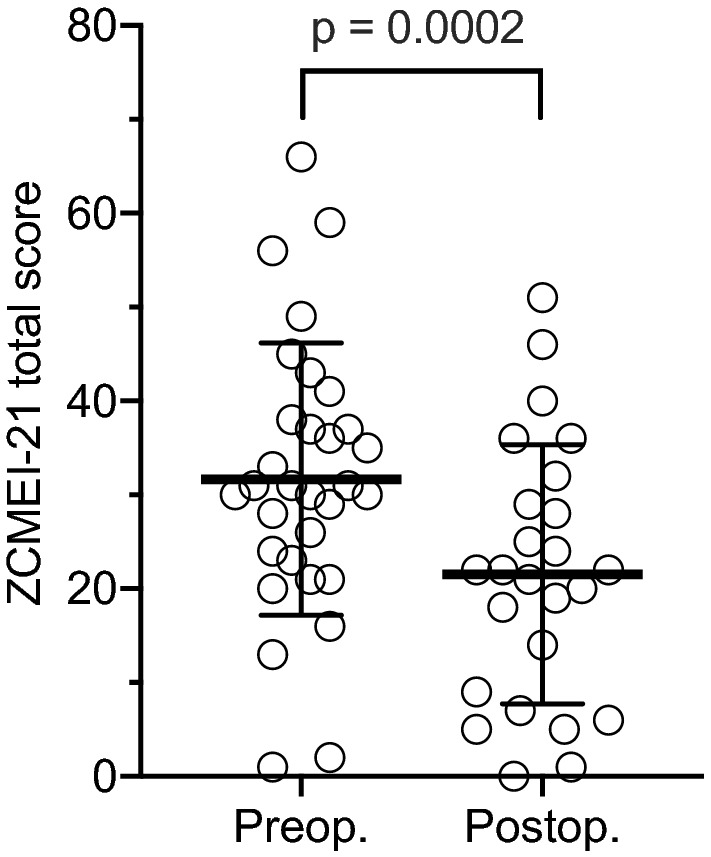
Pre- and postoperative ZCMEI-21 total scores. A lower ZCMEI-21 total score corresponds to a better HRQoL. The bold line represents the mean; error bars indicate standard deviation

**Fig. 3:**
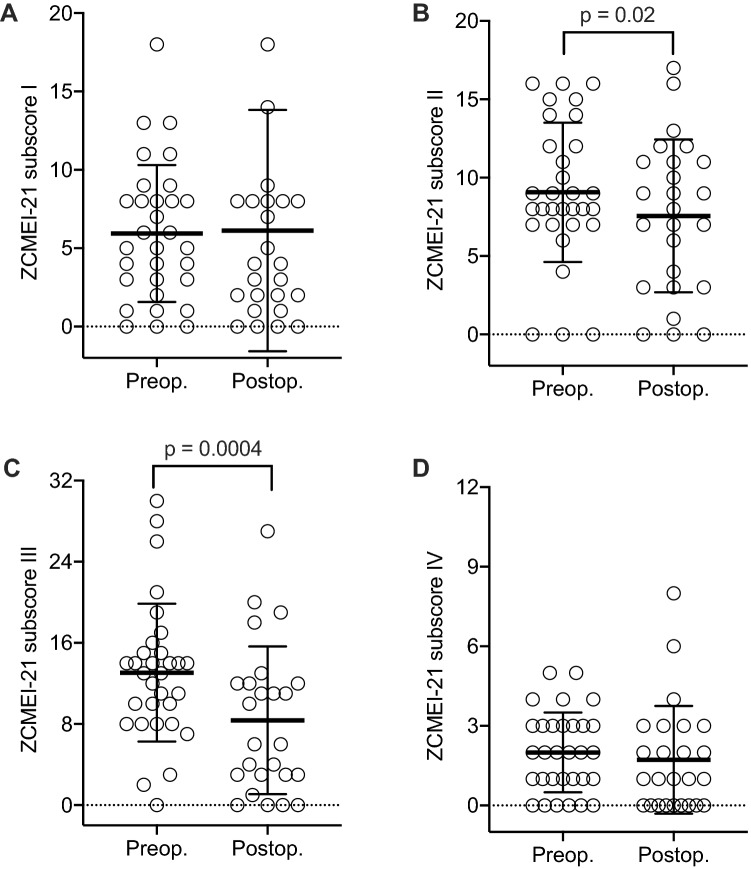
Changes in the ZCMEI-21 subscores. Subscores include subscore I [ear signs and symptoms; (**a**)], subscore II [hearing; (**b**)] subscore III [psychosocial impact; (**c**)], and subscore IV [medical resources; (**d**)]. A lower ZCMEI-21 total score corresponds to a better HRQoL. The bold line represents the mean; error bars indicate standard deviation

**Fig. 4:**
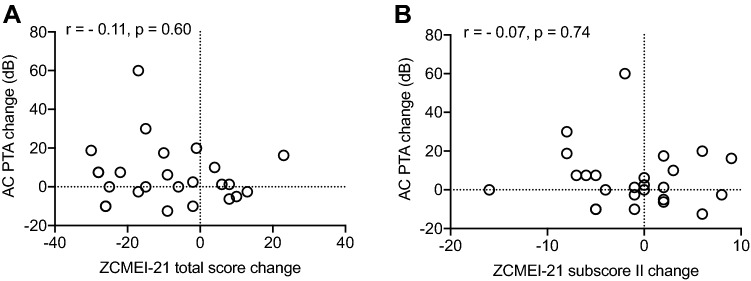
Scatter plot showing the association between the ZCMEI-21 total score (**a**) and hearing subscore (**b**) changes and the AC PTA change

The ZCMEI-21 total score changed from 31.7 (SD = 14.5) preoperatively to 17.4 (SD = 15.1) postoperatively (mean difference: − 14.3; SD = 19.1; *p* = 0.0002; Fig. 2). The mean change of subscore I (ear signs and symptoms) was 0.2 (SD = 9.3; *p* = 0.93; Fig. 3a). Subscore II (hearing) showed a mean change of − 3.0 (SD = 6.5; *p* = 0.02; Fig. 3b), subscore III (psychosocial impact) of − 6.3 (SD = 8.9; *p* = 0.0004; Fig. 3c), and subscore IV (medical resources) of − 0.2 (SD = 2.4; *p* = 0.63; Fig. 3d). No correlation between the change of the ZCMEI-21 total score and the AC change as well as between the change of the ZCMEI-21 hearing subscore and the change of the AC threshold was observed (Fig. 4).

The patients with an improvement of hearing (> 10 dB) did not show a higher ZCMEI-21 score shift (mean: − 6.6; SD: 19.6) compared to the mean of the entire cohort. Patients with a mild-to-moderate hearing handicap of < 50 dB AC (*n* = 14; 56%) after surgery had a mean improvement of 7.2 dB AC (SD = 11.3 dB). The mean ZCMEI-21 score change of these patients was − 6.9 (SD = 16.4).

## Discussion

Mastoid cavity obliteration is performed to reduce symptoms such as caloric vertigo and otorrhea, and to improve patient’s HRQoL including the reduction in frequency of ENT specialist consultations [6, 31]. In this study, a significant and clinically important improvement in HRQoL in patients undergoing mastoid cavity obliteration is demonstrated.

To our best knowledge, this is the first study to prospectively report an improvement in disease-specific HRQoL in patients undergoing mastoid cavity obliteration. Dornhoffer et al. were the first to draw attention to this topic by studying a small patient sample in a retrospective setting [25]. An increase of quality of life after mastoid obliteration and restoration of the middle ear space with cartilage reconstruction of the tympanic membrane was reported. Quality of life was assessed using the GBI, which is a generic questionnaire that measures change in quality of life and yields no information on the current quality of life. Moreover, this questionnaire is not disease-specific, which renders the instrument less sensitive to specific signs and symptoms [32]. The GBI was also used in another small retrospective cohort study demonstrating improved quality of life in 10 out of 12 (83%) patients after mastoid cavity obliteration with autologous bone [26]. In contrast, Joseph et al. recently reported less favorable results in ten patients with only four (40%) patients experiencing an improvement after mastoid cavity obliteration [27]. As a side note, the use of autologous obliteration material is recommended in open mastoid cavity and a positive influence on HRQoL has been reported [33].

Since clinical audiometry is performed in 5 dB steps, a mean improvement in the AC of only 4 dB is not considered clinically relevant. When analyzing the patients with higher improvement of hearing (> 10 dB) or smaller hearing handicap (< 50 dB) after surgery even smaller improvements in the total ZCMEI-21 score were observed compared to the complete cohort. Thus, it is assumed that although an overall hearing improvement was observed, the improvement in HRQoL cannot be attributed to the hearing improvement.

In the present study, the pre- and postoperative HRQoL using a disease-specific instrument, i.e., the ZCMEI-21 [15] was prospectively evaluated. The ZCMEI-21 was originally designed for prospective investigations and has been applied successfully in clinical trials [11, 24, 34]. In our cohort, a mean preoperative ZCMEI-21 total score of around 32 points which indicates an at least moderately impaired HRQoL [15] was found. After surgery, the mean ZCMEI-21 total score decreased to around 17 points. This value corresponds to no or a slight impairment in HRQoL [15]. The observed change of the ZCMEI-21 total score in this study corresponds to a large clinically important change [24]. This change was independent from the hearing improvement, since no correlation between the AC threshold change and the questionnaire change was observed. Interestingly, the two subscores that showed the highest change were the subscore II (hearing) and subscore III (psychosocial impact). Therefore, it is assumed that primarily symptoms such as otorrhea and foetor, but not an impaired hearing, decrease HRQoL in symptomatic mastoid cavities. Furthermore, the applicability of hearing aids may be complicated by a symptomatic mastoid cavity and may also maintain a draining cavity. Taken together, the results of this study indicate a highly significant and clinically important improvement in HRQoL after mastoid cavity obliteration and consequently encourage this procedure. Moreover, the results are in line with previous studies, showing that the use of autologous material is safe and effective [6, 33]. It is highly recommended to use reconstructive measures during primary surgery. Large cavities during primary should be avoided. In small mastoids with little or no aeration, small stable cavities might be achievable if surgical principles are followed. These include a smooth cavity with a floor which is even to the outer ear canal (no facial ridge) enabling an epithelization of the cavity without retention. The meatal entrance must be in proportion to the cavity behind.

This study has several limitations. First, a relatively small cohort was studied, which may be explained by the fact that mastoid cavity creation during primary surgery has become less frequent. On the other hand, due to a lack of knowledge, patients with discharging cavities may not be provided with information about further surgical treatment options. Patients with dry cavities with only calorically triggered complaints may be reluctantly referred for revision surgery. However, this cohort is larger than all the cohorts in which quality of life has been retrospectively assessed after mastoid cavity obliteration in the past [25–27]. Second, this study may exhibit a selection bias, since only symptomatic patients underwent surgery and were included. Asymptomatic patients usually are not referred to hospital and may not profit from cavity obliteration. This bias may lead to an overestimation of the treatment efficacy. Yet, the results of this study corroborate the notion that—if properly indicated—mastoid cavity obliteration has a substantial impact on patient’s HRQoL.

## Conclusion

Mastoid cavity obliteration with autologous material leads to a highly significant and clinically important improvement of HRQoL. This improvement in HRQoL is not related to the hearing improvement. It can be concluded that in cases of a symptomatic mastoid cavity, surgical obliteration provides a substantial subjective benefit regarding HRQoL and, therefore, patients can be encouraged to undergo this surgical procedure.
